# Fine-scale genetic structure and *wolbachia* infection of *aedes albopictus* (Diptera: Culicidae) in Nanjing city, China

**DOI:** 10.3389/fgene.2022.827655

**Published:** 2022-08-30

**Authors:** Heng-Duan Zhang, Jian Gao, Dan Xing, Xiao-Xia Guo, Chun-Xiao Li, Yan-De Dong, Zhong Zheng, Zu Ma, Zhi-Ming Wu, Xiao-Juan Zhu, Ming-Hui Zhao, Qin-Mei Liu, Ting Yan, Hong-Liang Chu, Tong-Yan Zhao

**Affiliations:** ^1^ State Key Laboratory of Pathogen and Biosecurity, Beijing Institute of Microbiology and Epidemiology, Beijing, China; ^2^ Department of Disinfection and Vector Control, Jiangsu Provincial Center for Disease Control and Prevention, Nanjing, China

**Keywords:** *Aedes albopictus*, genetic structure, haplotype, microspatial, urban region, microsatellite loci, *wolbachia*

## Abstract

**Background:** *Aedes albopictus* is an indigenous primary vector of dengue and Zika viruses in China. *Wolbachia* is a gram-negative and common intracellular bacteria, which is maternally inherited endosymbionts and could expand their propagation in host populations by means of various manipulations. Compared with research on the dispersion of *Ae. albopictus* at the macrospatial level (mainly at the country or continent level), little is known about its variation and *Wolbachia* infection at the microspatial level, which is essential for its management. Meanwhile, no local cases of dengue fever have been recorded in the history of Nanjing, which implies that few adulticides have been applied in the city. Thus, the present study examines how the *Ae. albopictus* population varies and the *Wolbachia* infection status of each population among microspatial regions of Nanjing City.

**Methods:** The genetic structure of 17 *Aedes albopictus* populations collected from urban, urban fringe, and rural regions of Nanjing City was investigated based on 9 microsatellite loci and the mitochondrial *coxI* gene. The *Wolbachia* infection status of each population was also assessed with *Wolbachia* A- and *Wolbachia* B-specific primers.

**Results:** Nine out of 58 tested pairs of microsatellite markers were highly polymorphic, with a mean PIC value of 0.560, and these markers were therefore chosen for microsatellite genotyping analysis. The Na value of each *Ae. albopictus* population was very high, and the urban area populations (7.353 ± 4.975) showed a lower mean value than the urban fringe region populations (7.866 ± 5.010). A total of 19 *coxI* haplotypes were observed among 329 *Ae. albopictus* individuals via haplotype genotyping, with the highest diversity observed among the urban fringe *Ae. albopictus* populations (Hd = 0.456) and the lowest among the urban populations (Hd = 0.277). Each *Ae. albopictus* population showed significant departure from HWE, and significant population expansion was observed in only three populations from the urban (ZSL), urban fringe (HAJY), and rural areas (HSZY) (*p* < 0.05). Combined with DAPC analysis, all the *Ae. albopictus* populations were adequately allocated to two clades with significant genetic differences according to population structure analysis, and the best K value was equal to two. AMOVA results showed that most (96.18%) of the genetic variation detected in *Ae. albopictus* occurred within individuals (F_IT_ = 0.22238, *p* < 0.0001), while no significant positive correlation was observed via isolation by distance (IBD) analysis (*R* ^2^ = 0.03262, *p* = 0.584). The TCS network of all haplotypes showed that haplotype 1 (H1) and haplotype 4 (H4) were the most frequent haplotypes among all populations, and the haplotype frequency significantly increased from urban regions (36.84%) to rural regions (68.42%). Frequent migration was observed among *Ae. albopictus* populations from rural to urban regions via the urban fringe region, with four direct migration routes between rural and urban regions. Furthermore, *Wolbachia* genotyping results showed that most of the individuals of each population were coinfected with *Wolbachia* A and *Wolbachia* B. The independent infection rate of *Wolbachia* A was slightly higher than that of *Wolbachia* B, and no significant differences were observed among different regions.

**Conclusion:** In the microspatial environment of Nanjing City, the urban fringe region is an important region for the dispersion of *Ae. albopictus* populations between rural and urban areas, and *Wolbachia* A and *Wolbachia* B coinfection is the most common *Wolbachia* infection status in all *Ae. albopictus* populations among different regions.

## Background

*Aedes albopictus*, which is distributed in southern Chaoyang, Liaoning Province and to eastern Tianshui in Gansu Province, China, is an important vector of several arboviruses, including dengue virus (DENV), Zika virus (ZIKV), and chikungunya (CHIKV), in mainland China and many other tropical and subtropical countries (Baolin, 1990; Li et al., 2018; Kraemer et al., 2019). Considering the frequent outbreaks of dengue fever among southern regions of China during the past few years, *Ae. albopictus* is regarded as the major target of the current integrated mosquito control and vector management program because it is widespread among urban and rural regions of southern China. The proposal of new reliable mosquito prevention and control strategies, especially for monitoring the dynamic changes and dispersion of mosquito populations among urban and rural regions also seems extremely important.

Source reduction and space spraying policies are the current strategies for the control of *Aedes albopictus* in China (Baolin, 1990). As the main strategy for *Ae. albopictus* control, the goal of source reduction is to reduce the population density of *Ae. albopictus* and eliminate its breeding sites without adulticide application; this approach is implemented when there is no outbreak of dengue fever. The insecticide space spraying policy is implemented as a supplementary control method when dengue fever emerges (Baolin, 1990; Hongliang, 2013; Yan-de et al., 2016). Source reduction may cause habitat fragmentation because the flight distance of *Ae. albopictus* is limited to 500 m (Taissa PDS et al., 2018) and could influence gene flow. Space spraying may decrease the densities of adult mosquitoes and the numbers of individuals sensitive to the insecticide (KO et al., 2017). The control of *Ae. albopictus* by source reduction is a component of the National Hygienic City program, which focuses on the built-up area of cities. The rural-urban continuum is a buffer zone where space spraying could prevent adult mosquitoes from invading built-up areas (Hongliang, 2013; Yan-de et al., 2016). It is crucial to study the population genetic structure of *Ae. albopictus* in the built-up areas, the rural-urban continuum, and rural areas of a city to achieve precise integrated mosquito management at the fine scale.

To understand the population genetic structure and dynamics of the gene flow patterns in habitats with a source reduction status based on mosquito population genetics studies, microsatellites are more frequently employed as markers in population studies of recent evolutionary events among subpopulations within an individual species or among closely related species relative to other biological markers, such as isoenzymes, random amplified polymorphic DNA (RAPD), restriction fragment length polymorphism (RFLP), single-strand conformation polymorphisms (SSCPs), and single nucleotide polymorphisms (SNPs), because of their per-locus advantages and high rate of polymorphism (Feldman et al., 1997; Sciarretta et al., 2001; Tsitrone et al., 2001; PORRETTA et al., 2006; Chambers et al., 2007; Tabachnick, 2010; Karl et al., 2012; Beebe et al., 2013; Delatte et al., 2013). These markers have been successfully used in studying many important vector mosquitos, such as *Ae. albopictus*, *Aedes aegypti, Anopheles sinensis*, *Culex pipiens quinquefasciatus*, *Cx. pipiens pallens* and *Anopheles gambiae* (Fonseca et al., 1998; Ma et al., 2011; Gélin et al., 2016; Miles et al., 2017; Wilke et al., 2018; Carvajal et al., 2020a; Gao et al., 2021). Such efforts revealed the fine-scale population genetic structure of the dengue mosquito vector *Ae. aegypti* in Metropolitan Manila, in the Philippines, based on microsatellite analysis, in low genetic differentiation was observed across mosquito populations, and high gene flow and/or weak genetic drift was found among mosquito populations, implying the passive or long-distance dispersal capability of *Ae. aegypti,* possibly through human-mediated transportation (Carvajal et al., 2020b). The structure of the studied populations of *Ae. albopictus* revealed using 12 microsatellite loci was divided into two groups, with restricted gene flow between these groups and no evidence of isolation by distance in the city of São Paulo (Multini et al., 2019).

*Wolbachia* is a *rickettsia* microorganism that is widely found in arthropods and can cause cytoplasmic incompatibility (CI), resulting in the decreasing densities of the host (Zhou et al., 1998a). The CI of mosquitos caused by *Wolbachia* was first discovered in *Cx. pipiens* and was later found in mosquitos such as *Ae. albopictus* and *Armigeres subalbatus* (Hertig and Wolbach, 1924; Hertig, 1936; Johnson, 2015; Nugapola et al., 2017). Numerous studies have shown that most widespread *Ae. albopictus* populations are infected with *Wolbachia* strain A or B alone, or coinfection by these strains. CI caused by different types of *Wolbachia* strains can affect the offspring of widespread *Ae. albopictus* populations, which in turn hinders the genetic communication of *Ae. albopictus* populations and affect the natural population genetic structure of the species (Sinkins et al., 1995a; Sinkins et al., 1995b; Zhou et al., 1998b; Das et al., 2014). Thus, the detection of *Wolbachia* infections in natural *Ae. albopictus* populations is another way to monitor the population dynamics of genetic structure.

Nanjing is an important tourism and economic centre in China and serves as an essential hub connecting the southern and northern regions of China via air transport, the Yangtze River, and railway transportation. *Ae. albopictus* is also the dominant mosquito in this city, and human-aided transmission is the main reason for its diffusion in natural conditions (Zhou et al., 1998b). Additionally, Nanjing is referred to as a National Hygienic City, and source reduction is carried out each year. The study of *Ae. albopictus* population genetics in Nanjing City to reveal the characteristics of genetic population structure provides a model of urban mosquito management-based source reduction and can contribute to an accurate approach for mosquito control. In the present study, a total of 17 *Ae. albopictus* habitats, distributed in either urban or rural areas, were investigated within the Nanjing region from July to September. Nine microsatellite loci in combination with the *wsp* gene were employed to analyse the genetic variability and *Wolbachia* infection status of each *Ae. albopictus* population. The results can provide a reference for subsequent mosquito management.

## Materials and methods

### Mosquito sampling

**FIGURE 1:**
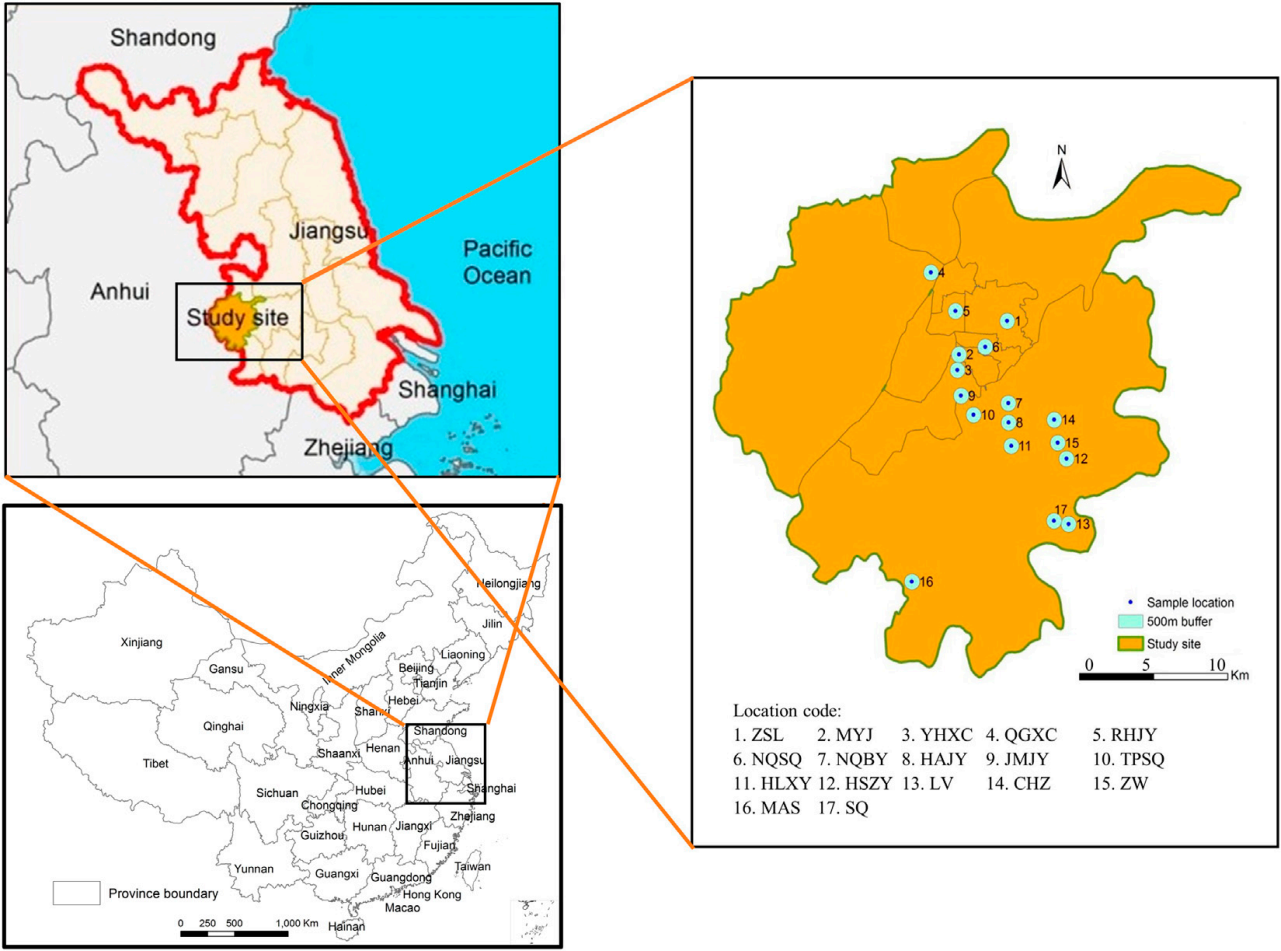
A province and county-level digital map was obtained from China Resource and Environment Science and Data Center (https://www.resdc.cn/), the detailed location of sample sites were mapped and showed using ArcGIS 10.7 (Environmental Systems Research Institute Inc., Redlands, CA, USA).

*Aedes albopictus* individuals were collected between July and September 2013 from 17 independent habitats within the Nanjing region, including urban, urban fringe and rural areas (Figure 1; Table 1). Larvae were surveyed using the container method (Zhang and Zeng, 2014); small indoor and outdoor water receptacles were examined for larvae at each sampling site. If larvae were found at a sampling site, a 1 km zone around that site was surveyed to confirm the essential interval between the different *Ae. albopictus* breeding habitats. Larvae were collected in the field with pipette and raised in a laboratory, and adults were identified as *Ae. albopictus* after emergence under a microscope (OLYMPUS, Japan) before DNA isolation (Lu, 2003).

**TABLE 1:** Location names, geographic coordinates and habitat type of all 17 *Ae. albopictus* sampling sites.

Sampling site	Code	Geographic co-ordinates	Habitat type
Zhongshanling	ZSL	32.058850N,118.850853E	Urban
Mingyangjie	MYJ	32.018052N,118.770455E	Urban
Yuhuaxincun	YHXC	31.999109N,118.767827E	Urban
Qiaogongxincun	QGXC	32.117827N,118.723619E	Urban
Renhejiayuan	RHJY	32.070897N,118.764664E	Urban
Nanqishequ	NQSQ	32.027401N,118.814241E	Urban
Ningquanbeiyuan	NQBY	31.958814N,118.852771E	Urban fringe
Henganjiayuan	HAJY	31.935296N,118.853283E	Urban fringe
Jingmingjiayuan	JMJY	31.967901N,118.773846E	Urban fringe
Taipingshequ	TPSQ	31.944673N,118.794716E	Urban fringe
Henglingxinyu	HLXY	31.906763N,118.857698E	Urban fringe
Hushuzhuyuan	HSZY	31.891234N,118.949898E	Rural
Lvyang	LV	31.811583N,118.953454E	Rural
Chunhuazhen	CHZ	31.938605N,118.929290E	Rural
Zhouwang	ZW	31.910558N,118.935393E	Rural
Maanshan	MAS	31.741411N,118.691826E	Rural
Shangqiao	SQ	31.815531N,118.929090E	Rural

### Analysis of breeding habitat buffer zone

We used GPS Pathfinder Office 3.10 to differentially correct the coordinates of *Aedes albopictus* breeding sites recorded with hand-held GPS, and the converted data were exported to an ESRI Shapefile. We used ArcGIS to delineate a 500 m radius buffer zone around each sampling site and to calculate the distance between each pair of sampling sites.

### Genotyping of mosquito samples

We genotyped a total of 510 mosquitoes, including 30 specimens (half male and female) from each of the 17 sample sites. The DNA of individual mosquitoes was extracted following the steps indicated in a TAKARA® DNA kit and stored in a -40 °C freezer until required. Individuals were genotyped based on the variation at nine microsatellite loci (PORRETTA et al., 2006; Chambers et al., 2007; Beebe et al., 2013; Delatte et al., 2013) (Table 2). Approximately 1–2 μL of each PCR product was diluted with 10 μL of autoclaved distilled water for DNA genotyping. Two microlitres of the diluted product was then added to 7.75 μL of Hi-Di™ Formamide and 0.25 μL of the Gene Scan 500 LIZ™ size standard. The mixture was heated at 94 °C for 5 min and then immediately chilled on ice for 2 min. Genotyping was carried out in a Genetic Analyzer 3130 xl (Applied Biosystems, United States). According to Bonacum J *et al.* (Bonacum et al., 2001), *coxI* sequence polymorphisms were investigated among 329 individuals. Briefly, the DNA amplification of a 550-bp fragment of *coxI* was performed on a T100 Thermal Cyclerin a 50 µL reaction mix containing 10 µL PCR buffer, 4 µL dNTPs, 1 µL primers and 0.5 µL PrimeSTAR® HS DNA polymerase (TAKARA, Japan). The PCR amplification program was set as follows: predenaturation at 94°C for 3 min, followed by 35 cycles of denaturation at 94°C for 30 s, annealing at 54°C for 45 s and elongation at 72°C for 1 min, with a final extension at 72°C for 10 min. All the PCR products were detected and separated by 2% agarose gel electrophoresis. The *coxI* target fragments were then cut from the gel under UV light and purified with the GenElute™ PCR Clean-Up Kit (TAKARA, Japan). Each purified PCR product was subsequently cloned into the pCR™2.1 vector with a TA Cloning™ Kit (TAKARA, Japan) and selected by bacterial liquid PCR with T7 promoter primers. Finally, at least 20 positive clones for each PCR product were subjected to the sequencing of both strands using an ABI 3730XL automatic sequencer (Applied Biosystems, United States).

**TABLE 2:** Microsatellite primers and *Wolbachia* detection primers used to genotype *Ae. albopictus* specimens sampled from different regions of Nanjing City.

Locus	Forward/Reverse sequence	Number of alleles	PIC	References
AealbA9	5′-TGG​GAC​AAG​AGC​TGA​AGG​AT-3’/5′-CTC​GTT​CTC​TAC​TCT​CTC​CGT​T-3’	41	0.819	Sciarretta et al. (2001)
AealbB52	5′-GGG​TCT​AGA​AGT​AAT​AGC​GAT​G-3’/5′-GCA​TTC​TTT​GCT​TCT​GTT​TGC-3’	12	0.276	Sciarretta et al. (2001)
AealbB51	5′-TCC​ACG​TGG​TAT​AAC​TCT​GA-3’/5′-GTA​GTT​GTC​CAA​TTA​ACA​TCG-3’	9	0.039	Sciarretta et al. (2001)
AEDC	5′-TGC​AGG​CCC​AGA​TGC​ACA​GCC-3’/5′-TCC​GCT​GCC​GTT​GGC​GTG​AAC-3’	31	0.486	Karl et al. (2012)
Alb222	5′-GAC​GAG​AAC​GGT​GAA​CAG-3’/5′-GTC​GAA​GGT​ACA​AAT​AGA​TCG-3’	5	0.458	Chambers et al. (2007)
Albtri20	5′-GTG​CCG​TTG​ATC​ATC​CTG​TC-3’/5′-TCC​AGC​ACC​GTG​AGT​AAT​CC-3’	25	0.777	PORRETTA et al. (2006)
Albtri25	5′-CCA​ACC​AAC​AAC​CCA​GGA​AC-3’/5′-TAC​GAT​GCG​CAA​CCA​TCA​TC-3’	34	0.785	Nigel W. *et al.* (PORRETTA et al., 2006)
Albtri4	5′-TGG​CGA​CCT​ATT​ATA​CCC​GC-3’/5′-CAA​CTC​GTT​CCT​TGA​CCG​TG-3’	16	0.611	PORRETTA et al. (2006)
Albtri18	5′-ACA​CAA​TTG​CCG​TTC​AGC​TC-3’/5′-CGT​CTA​ATA​GCT​CCG​GTC​CC-3’	43	0.789	PORRETTA et al. (2006)
*coxI*	5′-GGA​GGA​TTT​GGA​AAT​TGA​TTA​GTT​C-3′/5′-CCC​GGT​AAA​ATT​AAA​ATA​TAA​ACT​TC-3′	—	—	Bonacum et al. (2001)
*Wolbachia* Type A	5′-CCA​GCA​GAT​ACT​ATT​GCG-3’/5′-AAA​AAT​TAA​ACG​CTA​CTC​CA-3’	—	—	Zhou et al. (1998a)
*Wolbachia* Type B	5′-AAG​GAA​CCG​AAG​TTC​ATG-3’/5′-AAA​AAT​TAA​ACG​CTA​CTC​CA-3’	—	—	Zhou et al. (1998a)
*Wolbachia* Type A&B	5′-TGG​TCC​AAT​AAG​TGA​TGA​AGA​AAC-3’/5′-AAA​AAT​TAA​ACG​CTA​CTC​CA-3’	—	—	Zhou et al. (1998a)

### Data analysis

Genetic diversity (expected (He), observed (Ho) heterozygosity), the mean number of alleles (Na), and F-statistic parameters (F_IS_, F_ST,_ and F_IT_) were estimated from allele frequencies with FSTAT 2.9.3.2 (Goudet, 1995). For each locus-population combination in the global dataset and population groupings, we used Fisher’s exact test with Bonferroni correction to test for possible deviations from Hardy–Weinberg equilibrium (HWE) using POPGENE 1.32 (Rousset, 2008). Exact *p*-values were estimated using the Markov chain algorithm with 10,000 dememorizations, 500 batches, and 5000 iterations per batch. Pairwise differences between populations (Slatkin, 1995) and AMOVA analysis were displayed using Arlequin software 3.5.1.3 (Excoffier and Lischer, 2010) based on allelic frequency. In addition, the F_ST_ of each population was calculated from the mean value of the pairwise differences between populations. Bayesian clustering algorithms were implemented in STRUCTURE 2.3.3 (Pritchard et al., 2000; Falush et al., 2003) to infer population structure and explore the assignment of individuals and populations to specific genetic clusters. A UGPMA tree was built via NTsys software 2.10e. Excess heterozygosity at a significant number of loci is an indicator of a genetic bottleneck, whereas the converse situation (i.e., He, Heq) (Piry et al., 2017) may indicate population expansion. Estimates of Heq were computed using two mutation models for microsatellite evolution: a two-phase mutation model (T.P.M.) (Kimura and Ohta, 1978) and the stepwise mutation model (S.M.M.) (Nei, 1978). A Mantel test of the significance of genetic and geographic distances between each population was performed via the R package “adegenet 1.3”. Referring to the *CoxI* standard sequence MG198604.2, all the *CoxI* sequences were aligned with MAFFT 7.037 and credited via BioEdit 7.0.9.0. The haplotypes of all the populations were screened by DnaSP 6.0, and the TCS network was built via popART software 1.7, and the migration analysis was performed via R package “divMigrate” under R environment.

### Detection of *wolbachia* infection

Following the *Wolbachia wsp* gene classification method established by Zhou *et al.* (Zhou et al., 1998a), we used three pairs of diagnostic primers to detect and identify *Wolbachia*.81F is a universal primer that can amplify a fragment of approximately 590–632 bp in all known *Wolbachia* strains. 328F amplifies an approximately 380 bp fragment specific to *Wolbachia* Type A, whereas 183F amplifies an approximately 501 bp fragment specific to *Wolbachia* Type B. The volume of amplification reaction was 50 μL, which included 25 µL Taq DNA polymerase, 16 µL ddH_2_O, 2 µL 10 μmol/L, the upstream and downstream primers, and a 5 µL DNA template. The amplification conditions were as follows: 95°C predenaturation for 3 min, followed by 35 cycles of amplification at 94°C for 1 min, 55°C for 1 min, and 72°C for 1 min, and a final extension at 72°C for 7 min. Five microlitres of the above PCR product was used in 1.2% agarose gel electrophoresis. The results were examined under UV light, and the remaining products were sent to Tianyi Biotechnology Company Ltd.

## Results

### Genetic diversity and population structure based on microsatellite analysis

In the present study, a total of 17 *Aedes albopictus* populations were collected from urban, urban fringe, and rural areas of Nanjing City (Table 1 and Figure 1). Each of the 17 breeding site buffer zones was isolated from each other, indicating the independence of each population. Additionally, 58 pairs of microsatellite markers were obtained from previous research, 9 of which were highly polymorphic and were therefore chosen for microsatellite genotyping analysis (Table 2). The PIC values of each locus ranged from 0.039 to 0.819, with a mean of 0.560. According to the definition of PIC values provided by Allah *et al.*, nearly all the selected markers were highly informative (PIC value >0.5); the exception was AaelbB51, which had a value <0.25 and was considered to be a marker with low informativeness (Table 2). The linkage disequilibrium (LD) test showed that 286 pairs of loci among a total of 2412 (16.15%) across all locations were in significant LD after Bonferroni correction, and no consistency was found among them.

As show in Table 3, the observed number of alleles (Na) in each *Aedes albopictus* population was very high. The mean Na value of the urban area populations (7.353 ± 4.975) was lower than that of the rural area populations (7.387 ± 4.908), while the Na values of these two areas were all lower than those of the urban fringe area, which was 7.866 ± 5.010. The observed heterozygosity (Ho) values of all areas ranged from 0.543 ± 0.030 to 0.570 ± 0.030, which were lower than the expected heterozygosity (He) values (ranging from 0.566 ± 0.094 to 0.606 ± 0.096). The F_IS_ value of each population ranged from −0.026 to 0.218, and each *Ae. albopictus* population showed significant departure from HWE (Table 3, Additional file 2: Supplementary Table S1). Pairwise F_ST_ analysis among different regions showed that the variations in the 17 populations were all significant and ranged from 0.003 to 0.092, and the F_ST_ value between urban and urban fringe regions (0.011) was greater than the F_ST_ value between the urban and rural regions (0.009) and the F_ST_ value between urban fringe and rural regions (0.007) (Additional file 3: Supplementary Table S2). Heterozygosity tests of all 17 *Ae. albopictus* populations based on the stepwise mutation model (S.M.M.) revealed that only three populations from the urban (ZSL), urban fringe (HAJY), and rural areas (HSZY) displayed significant bottleneck effects (*p*-values < 0.05), while four more populations from the urban (YHXC and NQSQ), urban fringe (TPSQ), and rural areas (CHZ) were also observed to have significant bottleneck effects under the two-phase model (T.P.M.) (Additional file 4 Supplementary Table S3).

**TABLE 3:** Polymorphism and Inbreeding coefficient analysis at nine microsatellite loci in 17 *Ae. albopictus* specimens collected from 17 populations in Nanjing, China.

Population^a^	Sample size	Na	Ho	He	F_IS_
ZSL	30	8.000 ± 5.120	0.536 ± 0.031	0.595 ± 0.096	0.092^b^
MYJ	30	5.780 ± 3.490	0.474 ± 0.030	0.541 ± 0.106	0.127^b^
YHXC	30	7.560 ± 4.800	0.570 ± 0.030	0.604 ± 0.104	0.058
QGXC	30	8.000 ± 5.890	0.607 ± 0.030	0.622 ± 0.103	0.022
RHJY	30	7.000 ± 5.170	0.582 ± 0.030	0.605 ± 0.091	0.035
NQSQ	30	7.780 ± 5.380	0.604 ± 0.030	0.635 ± 0.085	0.047
**Mean**	30	**7.353 ± 4.975**	**0.562 ± 0.030**	**0.600 ± 0.097**	**—**
NQBY	30	7.000 ± 5.100	0.515 ± 0.030	0.551 ± 0.112	0.063
HAJY	30	8.670 ± 4.740	0.493 ± 0.030	0.630 ± 0.075	0.218^c^
JMJY	30	7.890 ± 5.440	0.555 ± 0.030	0.648 ± 0.093	0.142^c^
TPSQ	30	9.440 ± 5.530	0.590 ± 0.030	0.656 ± 0.099	0.090^b^
HLXY	30	6.330 ± 4.240	0.563 ± 0.030	0.566 ± 0.094	0.005
**Mean**	30	**7.866 ± 5.010**	**0.543 ± 0.030**	**0.566 ± 0.094**	**—**
HSZY	30	7.330 ± 4.500	0.537 ± 0.030	0.583 ± 0.096	0.076^b^
LV	30	7.440 ± 5.610	0.541 ± 0.030	0.605 ± 0.112	0.104^b^
CHZ	30	8.330 ± 5.430	0.537 ± 0.030	0.626 ± 0.099	0.148^c^
ZW	30	6.890 ± 4.760	0.596 ± 0.030	0.583 ± 0.099	−0.026
MAS	30	7.220 ± 4.470	0.596 ± 0.030	0.630 ± 0.085	0.050
SQ	30	7.110 ± 4.680	0.612 ± 0.030	0.608 ± 0.087	−0.007
**Mean**	**30**	**7.387 ± 4.908**	**0.570 ± 0.030**	**0.606 ± 0.096**	**—**

a See Figure 1 for geographic location.

b *p* < 0.05

c :*p* < 0.01.

The bold indicates the mean genetic value of 5~6 locations.

**FIGURE 2:**
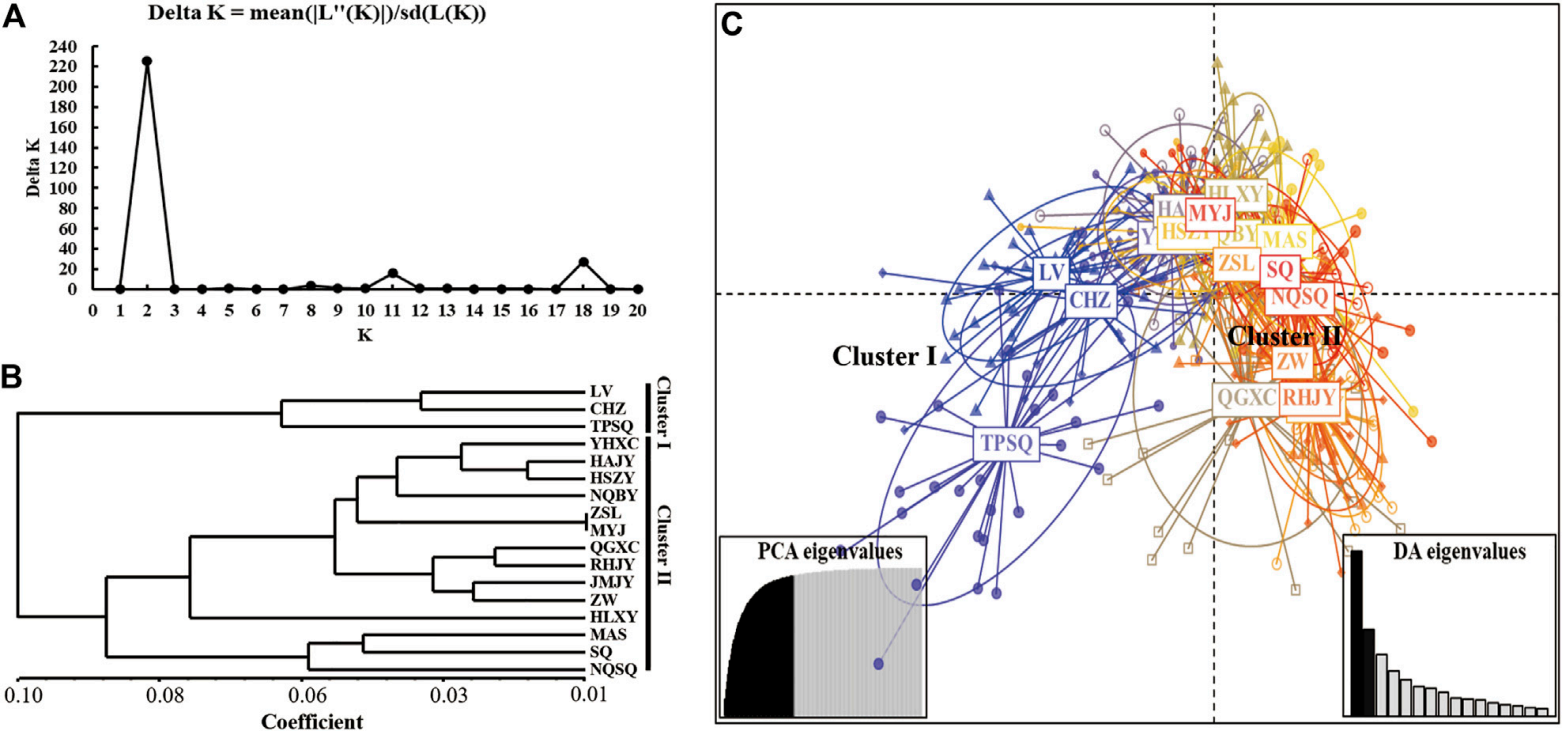
The population structure analysis of all 17 *Ae. albopictus* populations based on 9 microsatellite loci. **(A)** K values assessed via Evanno *et al.*‘s ΔK methods; **(B)** Bayesian clustering analysis of all *Ae. albopictus* populations; **(C)** DAPC analysis of all *Ae. albopictus* populations, and 86.4% of variation was explained by 50 PCs.

**FIGURE 3:**
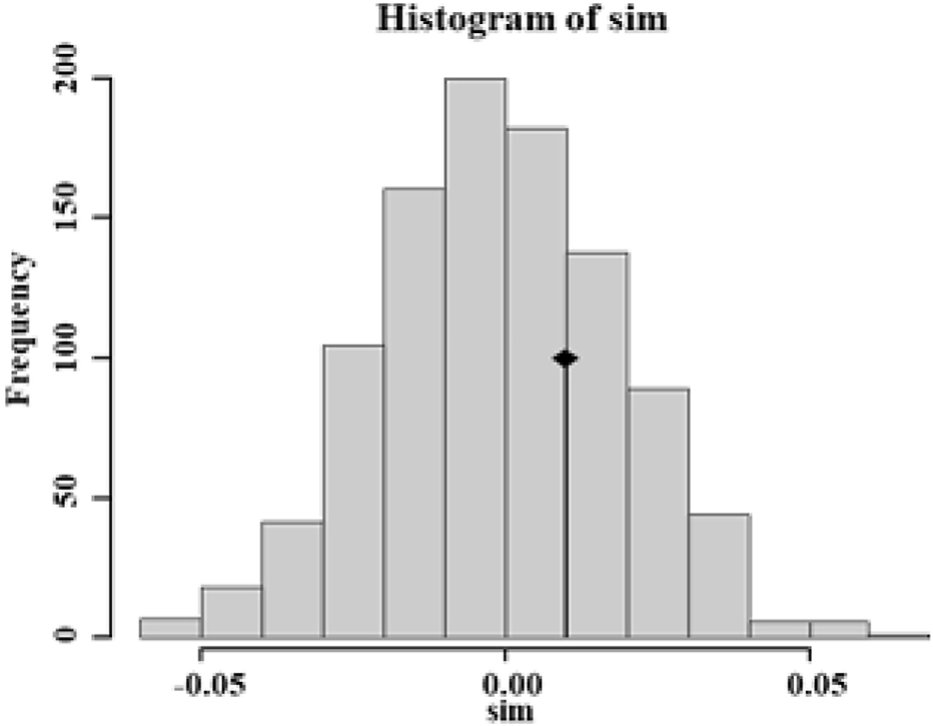
Isolation by distance (IBD) analysis of all 17 *Ae. Albopictus* populations from urban, urban fringe, and rural areas of Nanjing City.

According to the population structure analysis results, all *Aedes albopictus* populations were adequately allocated to two clades with significant genetic differences, and the best K value, as determined via the *ΔK* method of Evanno *et al.*, was also equal to two (Figures 2A,B). Additionally, a total of 88.7% of the variation was explained by 100 PCs in the discriminant analysis of principal components (DAPC) analysis; the structure analysis results revealed two genetically isolated groups (Figure 2C). The AMOVA results are presented in Table 4. From the total genetic variation partitioned, 5% could be attributed to differences among individuals within populations and 95% to differences within individuals (F_IS_ = 0.02171, F_IT_ = 0.22238, and all *p* < 0.0001). In contrast to the high individual variation observed, no significant positive correlation was observed among all individuals via isolation by distance (IBD) analysis (*R* ^2^ = 0.03262, *p* = 0.584) (Figure 3).

**TABLE 4:** AMOVA test of all 17 *Ae. albopictus* populations collected from different regions of Nanjing, China.

Source of variation	d.f	Sum of squares	Variance of components	Percentage	*p* Value	Fixation indices
Among groups	16	367.478	0.20129 Vb	5	*p* < 0.0001	F_SC_ = 0.07025
Among individuals within groups	493	2191.127	0.05783 Vc	42	*p* < 0.0500	F_IS_ = 0.02171
Within individuals	510	2171.000	2.60624 Vd	53	*p* < 0.0001	F_IT_ = 0.22238

### Haplotype diversity and network analysis based on coxI sequences

A total of 19 *coxI* haplotypes were observed among 329 *Aedes albopictus* individuals based on an analysis of *coxI* sequences, and the haplotype indices changed dramatically across *Ae. albopictus* populations from different area (Table 5). The haplotype diversity (Hd) ranged from 0.0909 (RHJY, urban region) to 0.6727 (JMJY, urban fringe region), with nucleotide diversity (*π*) ranging from 0.0002 (RHJY, urban region) to 0.0024 (JMJY, urban fringe region). The average number of nucleotide differences (k) ranged from 0.0909 (RHJY, urban region) to 1.1636 (JMJY, urban fringe region), and the number of polymorphic sites ranged from 1 to 9 across all the populations. Overall, the urban fringe *Ae. albopictus* populations showed the highest diversity, with mean Hd, π, and k values of 0.4562, 0.0018 and 0.8836, respectively, whereas the urban populations showed the lowest diversity.

**TABLE 5:** The genetic diversity indices of all 17 *Aedes albopictus* populations via Mitochondrial *coxI* gene.

No.	SS	Region	h	Hd	k	π	s	Tajima’s D	Details
1	RHJY	Urban	2	0.0909	0.09091	0.00018	1	−1.1624	H1(21); H2(1)
2	ZSL	Urban	4	0.2842	0.3	0.00064	3	−1.72331	H1(17); H2(1); H3(1); H4(1)
3	MYJ	Urban	4	0.4316	0.46842	0.00095	3	−1.19135	H1(15); H2(3); H3(1); H4(1)
4	YHXC	Urban	4	0.3632	0.46842	0.00096	3	−1.19135	H1(16); H2(1); H3(1); H4(2)
5	QGXC	Urban	3	0.2157	0.22222	0.00045	2	−1.50776	H1(16); H2(1); H3(1)
6	NQSQ	Urban	1	NA	NA	NA	NA	NA	H1(2)
7	NQBY	Urban fringe	2	0.1053	0.94737	0.00195	9	−2.20521^a^	H1(18); H2(1)
8	HAJY	Urban fringe	1	NA	NA	NA	NA	NA	H1(10)
9	JMJY	Urban fringe	4	0.6727	1.16364	0.00241	3	0.46577	H1(6); H2(3); H3(1); H4(1)
10	TPSQ	Urban fringe	4	0.3801	0.60819	0.00125	4	−1.37975	H1(1); H2(1); H3(15); H4(2)
11	HLXY	Urban fringe	5	0.6667	0.81522	0.00168	4	−0.65346	H1(11); H2(9); H3(2); H4(1); H5(1)
12	HSZY	Rural	2	0.2637	0.26374	0.00054	1	−0.34144	H1(12); H2(2)
13	LV	Rural	4	0.308	0.32609	0.00067	3	−1.49431	H1(20); H2(1); H3(2); H4(1)
14	CHZ	Rural	3	0.5229	0.57516	0.00117	2	−0.02647	H1(4); H2(12); H3(2)
15	ZW	Rural	4	0.3983	0.42857	0.00087	3	−1.23957	H1(17); H2(3); H3(1); H4(1)
16	MAS	Rural	5	0.2698	0.28571	0.00067	4	−1.88915^b^	H1(24); H2(1); H3(1); H4(1); H5(1)
17	SQ	Rural	4	0.4167	0.45333	0.00096	3	−1.06644	H1(19); H2(3); H3(1); H4(2)

a *p* < 0.01

b *p* < 0.05.

**FIGURE 4:**
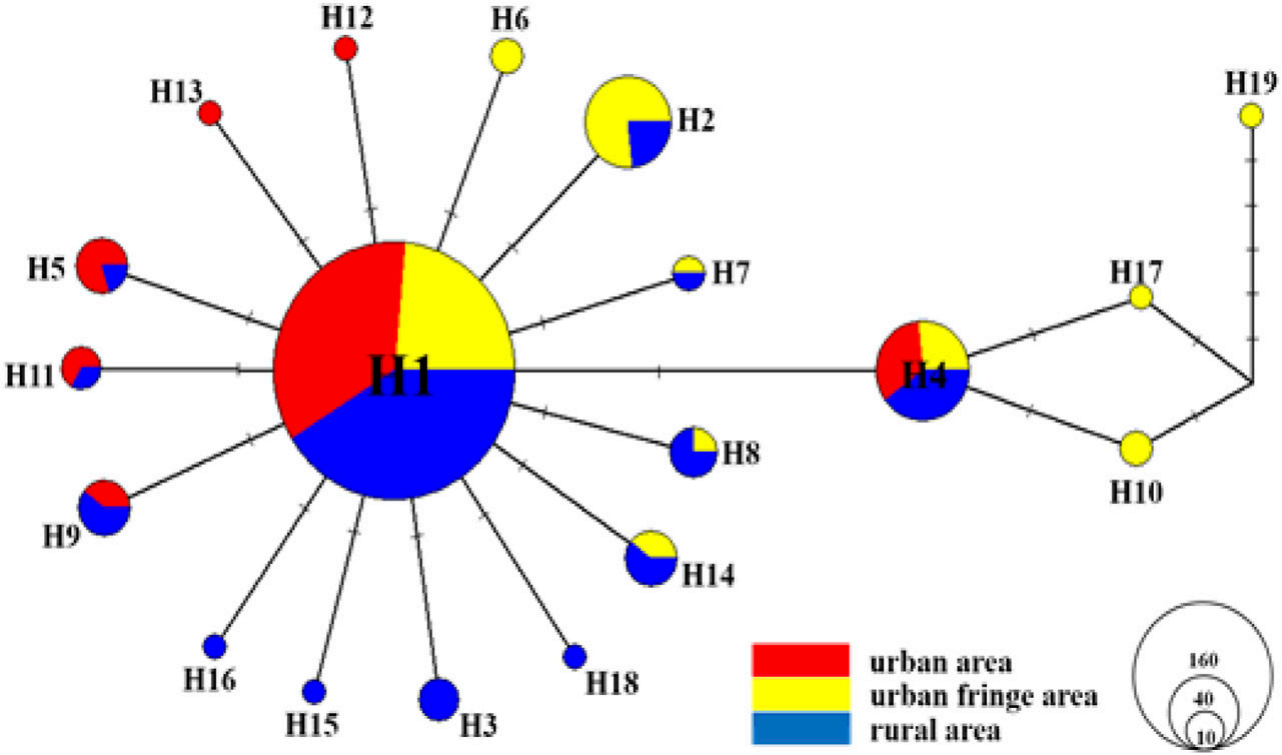
TCS haplotype network for the *coxI* gene of all *Ae. albopictus* individuals (*n* = 329) from urban, urban fringe, and rural areas of Nanjing City. The sizes of circles are proportional to haplotype frequency, and each line segment represents a single mutation.

As shown in Figure 4, a TCS network was reconstructed with all 329 *coxI* sequences, and the results showed that haplotype 1 (H1) and haplotype 4 (H4) were the most frequent haplotypes and were distributed equally among all three sampling regions. Nearly all the other haplotypes were derived from H1 with one or more mutations, and the haplotype frequency increased significantly from the urban region (36.84%) to the rural area (68.42%). Relative to the urban regions (only H12 and H13), more exclusive haplotypes were detected among urban fringe regions (H6, H10, H17, and H19) and rural regions (H3, H15, H16, and H18). No additional shared haplotypes were detected between rural and urban fringe regions except H1 and H4.

### Migration analysis and *wolbachia* genotyping

**FIGURE 5:**
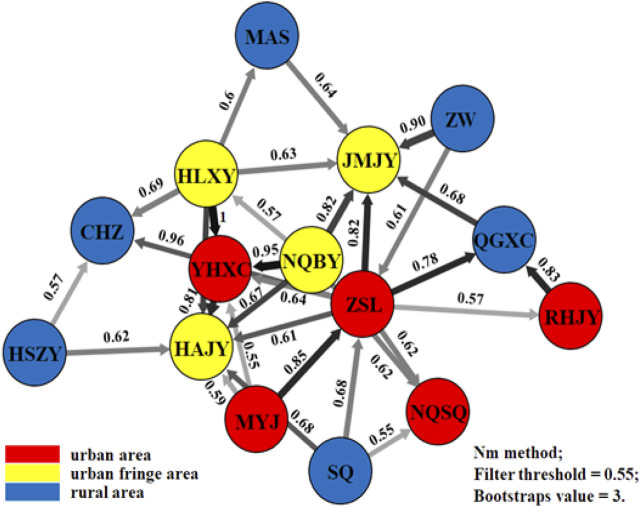
Migration patterns of *Ae. albopictus* among urban, urban fringe, and rural areas of Nanjing City.

Migration patterns were assessed using divMigrate networks representing all 17 *Aedes albopictus* populations. As expected, *Ae. albopictus* was observed to migrate frequently from rural to urban regions via the urban fringe region, and four direct migration routes between rural and urban regions were observed (between SQ and ZSL, ZSL and QGXC, SQ and NQSQ, and RHJY and QGXC, with Nm values as high as 0.68, 0.78, 0.55, and 0.83, respectively) (Figure 5). The obtained Tajima’s D values were negative, no statistically significant *p*-values were obtained except in population JMJY (urban fringe region), and the unimodal mismatch distributions were observed mainly among the urban fringe *Ae. albopictus* populations (Additional file 1: Supplementary Figure S1).

**FIGURE 6:**
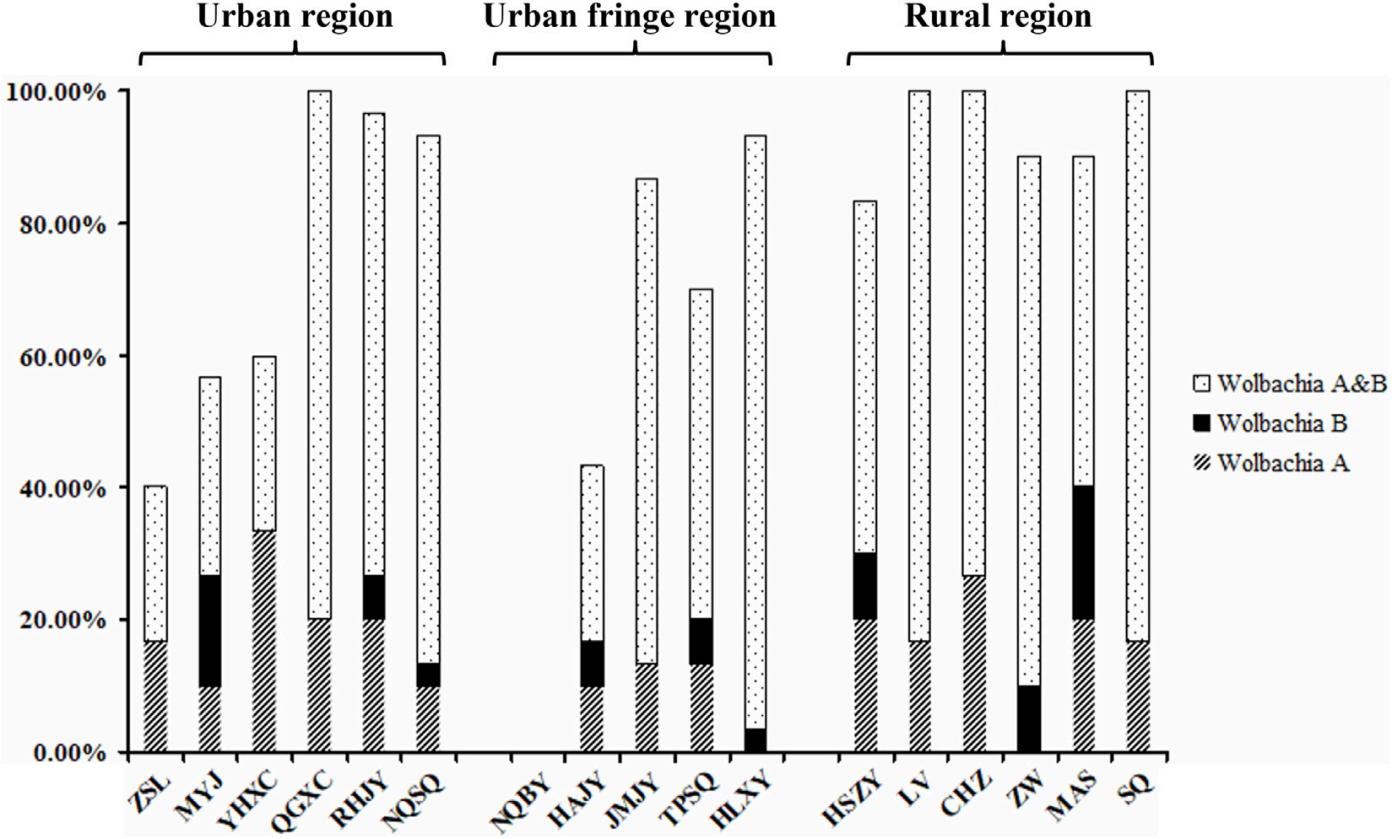
Independent infection rate of *Wolbachia* A and *Wolbachia* B among different *Ae. albopictus* populations from urban, urban fringe, and rural areas of Nanjing City.

Fragments of 500 bp and 380 bp were amplified from each of the 30 samples from all *Aedes albopictus* populations with the *Wolbachia* A- and *Wolbachia* B-specific primers, respectively, confirming *Wolbachia* infection. The sequencing results showed that most of the individuals of each population were coinfected with *Wolbachia* A and *Wolbachia* B, with infection rates (IR) ranging from 23.33% (ZSL, urban region) to 90.00% (HLXY, urban fringe region), and the mean infection rate of the rural region (70.56%) was significantly higher than that of the urban and urban fringe regions, which were 51.67 and 48.00%, respectively. Compared with the coinfection indices of *Wolbachia* A and *Wolbachia* B, the values of *Wolbachia* A and *Wolbachia* B for all individuals were low; the independent infection rate of *Wolbachia* A was slightly higher than that of *Wolbachia* B, and no significant differences were observed among different regions (Figure 6).

## Discussion

The number of alleles, heterozygosity, and polymorphism information content (PIC) are common indices for evaluating the genetic diversity of populations (Nei et al., 1975; Botstein et al., 1980; Maudetr et al., 2002). The number of alleles is generally positively related to the level of genetic diversity among individual mosquitoes from the same population. Heterozygosity is also a measure of the genetic diversity of a population; the higher population heterozygosity is, the greater genetic diversity, polymorphism levels and the potential variation available for natural selection to act on will be. PIC is a relatively good index of gene fragment polymorphism. PIC values >0.5 indicate highly polymorphic loci. In earlier research, microsatellite loci and *coxI* have been widely used to evaluate the genetic variation and population structure of mosquitoes at a fine scale. The average number of alleles in this study was 7.387, the average expected heterozygosity was 0.606, and the average polymorphic information content was 0.560, which also indicated a similar status of *Ae. aegypti* in several areas. (Chambers et al., 2007; Beebe et al., 2013; Delatte et al., 2013; Monteiro et al., 2014; Multini et al., 2019; Carvajal et al., 2020a; Regilme et al., 2021). The variation of these nine microsatellites indicated that the 17 *Ae. albopictus* populations presented a relatively rich PIC and relatively even gene frequency distribution in Nanjing. This approach was shown to be a highly effective and reliable way of assessing genetic diversity. The results also indicated that our sample size was more than adequate. The analysis of the *coxI* polymorphism data of *Ae. albopictus* showed different haplotypes in each population; 19 haplotypes were found among the 329 samples, and the mean haplotype diversity was 0.359, which suggested relatively high genetic diversity.

A bottleneck effect will lead to excess heterozygosity in a population (Nei et al., 1975). It was shown herein that the Nanjing populations of *Ae. albopictus*, including ZSL, YHXC, NQSQ, HAJY, TPSQ, HSZY, and CHZ, may have experienced a bottleneck effect based on the results of the S.M.M. and T.P.M. model. F_IS_ is an index of inbreeding within populations, where significant positive values indicate serious inbreeding within a population resulting in a loss of heterozygosity and significant negative values indicate outbreeding and an associated surplus of heterozygotes. The F_IS_ values were all positive for the ZW and SQ populations, indicating that the degree of inbreeding in the populations was severe. The Ho values were lower than the He values except for the ZW and SQ populations, which indicated that the *Ae. albopictus* populations showed high gene consistency and suggested that the population might be experiencing inbreeding. The neutral test results for Tajima’s *D* showed that all populations except for NQSQ, HAJY, and JMJY presented negative values, suggesting that *Ae. albopictus* populations in Nanjing were selectively eliminated or were expanding (Tajima, 1989). The bottleneck effect and heterozygosity results showed that the *Ae. albopictus* populations in Nanjing were not in a state of expansion but were being selectively eliminated.

Implemented mosquito management (IMM) is aimed at reducing the density of mosquito species, which could change mosquito population genetics. An IMM program was effective in lowering the density of *Ae. aegypti* during an outbreak of dengue fever in Madeira in 2012, and a recent bottleneck effect of *Ae. aegypti* was simultaneously identified via heterozygosity tests (Seixas et al., 2019). The IMM of dengue vectors in China is focused on source reduction under conditions with no dengue cases. When the reported cases of dengue indicate an outbreak of the virus, the strategy of *Ae. albopictus* control involves both the chemical control of adult mosquitoes and source reduction (Hongliang, 2013). Nanjing is a national health city in China. Source reduction is a regular program applied every year to meet the standard of mosquito control in national health cities (General Administration of Quality Supervision IaQotPsRoC and Standardization Administration of China, 2011). Breeding sites of *Ae. albopictus* are regularly destroyed, the populations of *Ae. albopictus* are selectively eliminated, and the habitat of *Ae. albopictus* has become fragmented, which hinders gene flow and causes population inbreeding, leading to a bottleneck effect in some populations in Nanjing.

*Aedes albopictus* generally inhabits a home range with a distance of less than 50 m, and the flight distances of this species do not exceed 500 m (Hawley, 1988; Marini et al., 2010). Studies have shown that artificial landscapes such as urban roads are potential barriers to mosquito communication and hinder gene flow (Mondal et al., 2005; Gubler et al., 2010; Stoddard et al., 2019; Carvajal et al., 2020b; Regilme et al., 2021). However, urbanization substantially increases mosquito density and larval development. The Various populations in urban, suburban, and rural areas show differences in genetic differentiation, and the population in rural areas is more differentiated than other populations based on the results of F_ST_ analysis, which is different from the findings of Trinidad and Cairns et al. (Hemme et al., 2010; Schmidt et al., 2018; Regilme et al., 2021). Consistent with studies of *Ae. albopictus* conducted in Guangzhou (Li et al., 2014), our findings showed that although man-made landscapes such as urban roads are significantly more common in urban areas than in urban fringe and rural areas, urban areas are characterized by a higher human population density and more frequent human activities than urban fringe and rural areas, meaning that *Ae. albopictus* in urban areas still has more opportunities to feed on blood, mate, and reproduce. The genetic divergence index (F_ST_) is an essential measure of population genetic divergence. Values within the range of 0.05–0.15 indicate moderate deviation, and values <0.05 indicate slight deviation (Balloux and Lugon-Moulin, 2002). The variation of the 17 populations was in the range of 0.003–0.092, indicating only slight genetic divergence among the 17 populations studied. Additionally, pairwise F_ST_ analysis among different regions showed that the F_ST_ value between urban and urban fringe regions (0.011) was greater than the F_ST_ value between urban and rural areas (0.009) and the F_ST_ value between urban fringe and rural areas (0.007), which indicates that the mosquitoes in the city come mainly from the urban fringes. Proliferation within cities is greater than direct movement from rural areas to cities, and the exchange of mosquito populations between cities and urban-rural junctions is greater than that between rural areas and urban-rural junctions. This may be because compared to rural areas, the urban-rural fringe is the main area where urban living resources are distributed, where the flow of people and logistics is intensive, with continuous transport to the city. At the same time, the corresponding resources in the city are transferred to other areas through the urban-rural fringe. Dispersal within the city has also been verified by migration analysis. The migration analysis showed that compared with the exchange of mosquitoes between the other two regions, there were 4 main mosquito communication paths between the city and the urban-rural junction, and the gene flow value along each path was greater than 0.8. A program of IMM can affect the gene flow of populations through area effects and isolation effects and ultimately lead to a decrease in the genetic diversity of populations. However, the results showed that 17 *Ae. albopictus* populations experienced genetic exchange, and there was no apparent loss of genetic diversity. This may have been due to the influence of the hosts, various vehicles and potential mates that different populations of *Ae. albopictus* may encounter.

Most populations of *Aedes albopictus* worldwide have been found to be infected with either *Wolbachia* Type A or B bacterial symbionts (Zhou et al., 1998b). CI can cause different populations of *Ae. albopictus* to be infected with different types of *Wolbachia*, causing them to produce few or no offspring, resulting in abnormal free mating between different populations and inbreeding within each population (Caragata et al., 2016; Schmidt et al., 2018; Xia et al., 2019; Zheng et al., 2019). Except for the NQBY population, all other populations were positive for *Wolbachia*. *Wolbachia* A and B sequencing results showed differences in *Wolbachia* infection status within each population. The genetic differentiation between populations is not obvious because *Ae. albopictus* individuals that are either infected with the same type of *Wolbachia* or not infected with *Wolbachia* can mate freely.

## Conclusion

The present mosquito control strategy helps reduce the abundance and diversity of *Aedes albopictus* population, however, the active human-aided mosquito dispersion helps recover the *Ae. albopictus* population in a short period of time. Therefore, the continuous dynamic monitoring of *Ae. albopictus* population is important and will provide a solid foundation for the scientific prevention and control of *Ae. albopictus*.

## Data Availability

The raw data supporting the conclusion of this article will be made available by the authors, without undue reservation.

## Abbreviations

AMOVA test, Analysis of molecular variation test; *coxI*, Mitochondrial cytochrome c oxidase subunit 1; CI, Cytoplasmic Incompatibility; DAPC, Discriminant analysis of principal components; F_IS_, Inbreeding coefficient; F_ST_, Genetic differences among populations; F_IT_: Genetic differences within individuals; Hd, Haplotype diversity; He, Excepted heterozygosity; Heq, Expected average heterozygosity; Ho, Observed heterozygosity; HWE, Hardy Weinberg equilibrium; IBD, Isolation by distance; IR, Infection Rates; IMM, Implemented mosquito management; LD, Linkage disequilibrium; Na, Observed number of alleles; Nm, Number of migrants; PIC, Polymorphism Information Content; S.M.M., Stepwise Mutation Model; T.P.M , Two phase model.
