# Supplementary material for: Fine-scale genetic structure and wolbachia infection of aedes albopictus (Diptera: Culicidae) in Nanjing city, China
Source: Front Genet. 2022 Aug 30;13:827655. doi: 10.3389/fgene.2022.827655 (PMC9468874; doi:10.3389/fgene.2022.827655)
Supplement: Supplementary file 1 [file Table1.DOCX]

**Additional file 2: Table S1.** Conformity to the Hardy-Weinberg Equilibrium of variation at 9 microsatellite loci in 17 *Ae. albopictus* populations in Nanjing, China

| Population | AealbA9 | AealbB52 | AealbB51 | AEDC | Alb222 | Albtri20 | Albtri25 | Albtri4 | Albtri18 |
| --- | --- | --- | --- | --- | --- | --- | --- | --- | --- |
| ZSL | 0.0522 | 0.0000^**^ | 0.0003^**^ | 0.0329^*^ | 0.0000^**^ | 0.0435^*^ | 0.4114 | 0.0000^**^ | 0.155 |
| MYJ | 0.0463^*^ | 1 | - | 0.512 | 0.0000^**^ | 0.0000^**^ | 0.0237^*^ | 0.0000^**^ | 0.0000^**^ |
| YHXC | 0.1077 | 1 | - | 0.7936 | 0.0000^**^ | 0.9481 | 0.0000^**^ | 0.0000^**^ | 0.0005^**^ |
| QGXC | 0.1467 | 0.1624 | - | 0.3518 | 0.0000^**^ | 0.5953 | 0.1878 | 0.0002^**^ | 0.0041^**^ |
| RHJY | 0.0037^**^ | 0.0000^**^ | - | 0.3273 | 0.0000^**^ | 0.9666 | 0.0010^**^ | 0.0000^**^ | 0.0003^**^ |
| NQSQ | 0.4768 | 0.0000^**^ | - | 0.0735 | 0.0000^**^ | 0.7388 | 0.0366^*^ | 0.0000^**^ | 0.5201 |
| NQBY | 0.9777 | - | - | 0.172 | 0.0000^**^ | 0.0738 | 0.6055 | 0.0000^**^ | 0.0000^**^ |
| HAJY | 0.0020^**^ | 0.2034 | 0.0000^**^ | 0.1541 | 0.0000^**^ | 0.0255^*^ | 0.0000^**^ | 0.0000^**^ | 0.0000^**^ |
| JMJY | 0.2593 | 0.0001^**^ | - | 0.0053^**^ | 0.0000^**^ | 0.3709 | 0.0010^**^ | 0.0000^**^ | 0.0319^*^ |
| TPSQ | 0.137 | 0.0070^**^ | - | 0.1254 | 0.0000^**^ | 0.3088 | 0.0000^**^ | 0.0000^**^ | 0.0000^**^ |
| HLXY | 0.1001 | 0.5519 | - | 0.3043 | 0.0000^**^ | 0.0034^**^ | 0.3645 | 0.0000^**^ | 0.0014^**^ |
| HSZY | 0.228 | 0.0300^*^ | - | 0.1257 | 0.0000^**^ | 0.0016^**^ | 0.0128^*^ | 0.0000^**^ | 0.0131^*^ |
| LV | 0.0000^**^ | 1 | - | 0.1495 | 0.0000^**^ | 0.0015^**^ | 0.0266^*^ | 0.0000^**^ | 0.0234^*^ |
| CHZ | 0.2097 | 0.1199 | 0.0539 | 0.5743 | 0.0000^**^ | 0.0687 | 0.0000^**^ | 0.0000^**^ | 0.0022^**^ |
| ZW | 0.5166 | 0.0000^**^ | - | 0.3883 | 0.0000^**^ | 0.3388 | 0.4328 | 0.0000^**^ | 0.0545 |
| MAS | 0.813 | 0.0000^**^ | 0.0166^*^ | 0.6514 | 0.0000^**^ | 0.0794 | 0.0301^*^ | 0.0000^**^ | 0.1196 |
| SQ | 0.0126^*^ | 0.0000^**^ | - | 0.6021 | 0.0000^**^ | 0.3324 | 0.177 | 0.0000^**^ | 0.0842 |
