# Supplementary figures and images for: Fine-scale genetic structure and wolbachia infection of aedes albopictus (Diptera: Culicidae) in Nanjing city, China
Source: Front Genet. 2022 Aug 30;13:827655. doi: 10.3389/fgene.2022.827655 (PMC9468874; doi:10.3389/fgene.2022.827655)

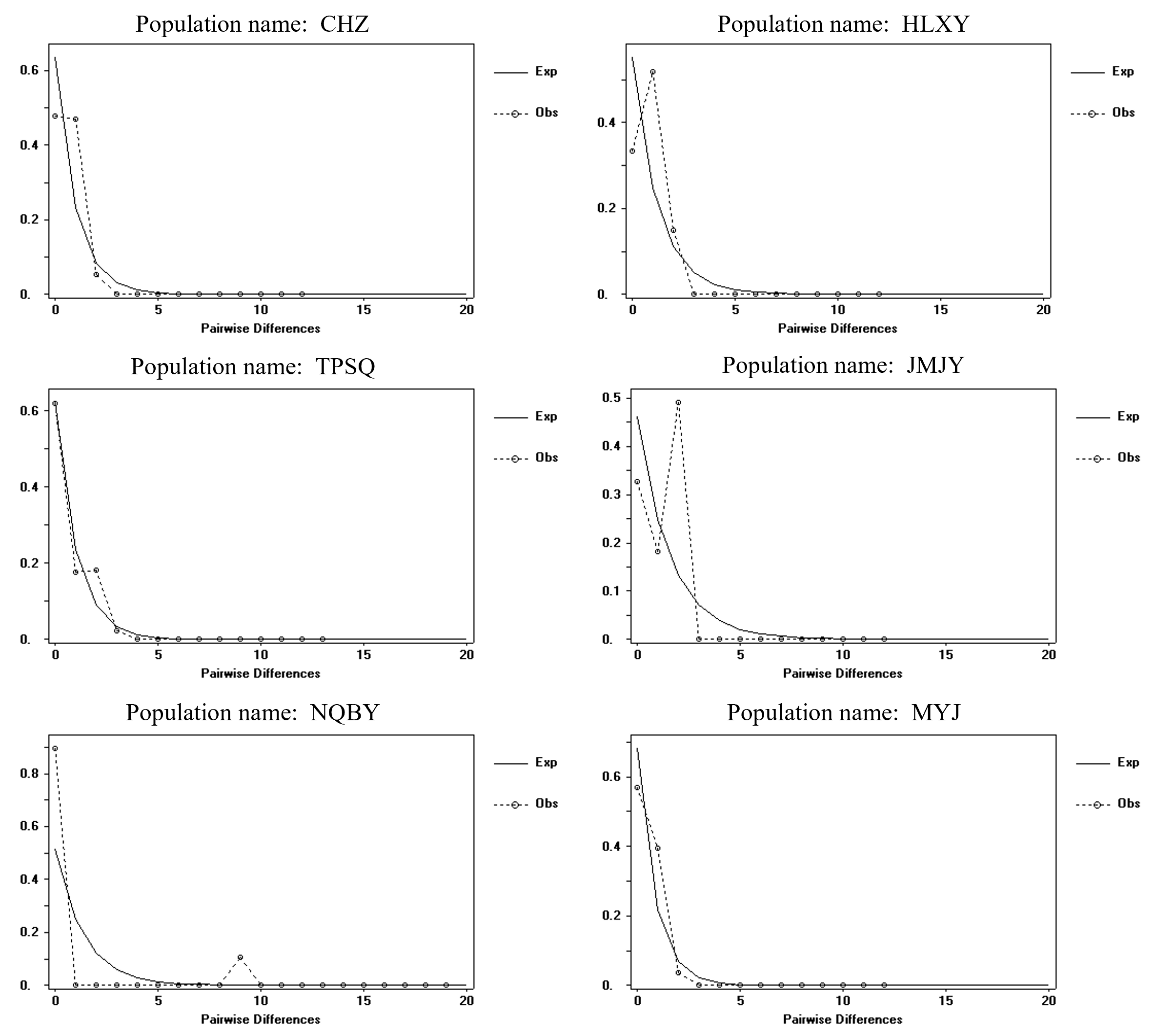

Supplement: Supplementary file 2 [file Image1.TIF]
