# Supplementary material for: Fine-scale genetic structure and wolbachia infection of aedes albopictus (Diptera: Culicidae) in Nanjing city, China
Source: Front Genet. 2022 Aug 30;13:827655. doi: 10.3389/fgene.2022.827655 (PMC9468874; doi:10.3389/fgene.2022.827655)
Supplement: Supplementary file 3 [file Table2.DOCX]

**Additional file 3: Table S2.** **Pair-wise genetic difference (*F_ST_*) (below diagonal) and geographic distance (above diagonal) among 17 *Aedes albopictus* populations**

|  | **ZSL** | **MYJ** | **YHXC** | **QGXC** | **RHJY** | **NQSQ** | **NQBY** | **HAJY** | **JMJY** | **TPSQ** | **HLXY** | **HSZY** | **LV** | **CHZ** | **ZW** | **MAS** | **SQ** |
| --- | --- | --- | --- | --- | --- | --- | --- | --- | --- | --- | --- | --- | --- | --- | --- | --- | --- |
| **ZSL** | 0.000 | + | + | + | + | + | + | + | + | + | + | + | + | + | + | + | + |
| **MYJ** | **0.003** | 0.000 | + | + | + | + | + | + | + | + | + | + | + | + | + | + | + |
| **YHXC** | **0.009** | 0.020 | 0.000 | + | + | + | + | - | + | + | + | + | + | + | + | + | + |
| **QGXC** | 0.014 | 0.030 | 0.016 | 0.000 | + | + | + | + | + | + | + | + | + | + | + | + | + |
| **RHJY** | 0.016 | 0.025 | 0.023 | **0.007** | 0.000 | + | + | + | - | + | + | + | + | + | + | + | + |
| **NQSQ** | 0.053 | 0.070 | 0.062 | 0.066 | 0.037 | 0.000 | + | + | + | + | + | + | + | + | + | + | + |
| **NQBY** | 0.030 | 0.037 | 0.023 | 0.039 | 0.037 | 0.084 | 0.000 | + | + | + | + | + | + | + | + | + | + |
| **HAJY** | 0.022 | 0.031 | **0.006** | 0.025 | 0.027 | 0.060 | 0.019 | 0.000 | + | + | + | + | + | + | + | + | + |
| **JMJY** | 0.022 | 0.041 | 0.018 | 0.015 | **0.008** | 0.042 | 0.031 | 0.025 | 0.000 | + | + | + | + | + | + | + | + |
| **TPSQ** | 0.028 | 0.037 | 0.023 | 0.027 | 0.041 | 0.073 | 0.058 | 0.034 | 0.032 | 0.000 | + | + | + | + | + | + | + |
| **HLXY** | 0.054 | 0.069 | 0.035 | 0.041 | 0.046 | 0.092 | 0.033 | 0.028 | 0.035 | 0.067 | 0.000 | + | + | + | + | + | + |
| **HSZY** | 0.029 | 0.035 | 0.019 | 0.033 | 0.030 | 0.071 | 0.026 | **0.007** | 0.029 | 0.039 | 0.041 | 0.000 | + | + | + | + | + |
| **LV** | 0.061 | 0.076 | 0.048 | 0.049 | 0.074 | 0.125 | 0.078 | 0.066 | 0.068 | 0.040 | 0.087 | 0.054 | 0.000 | + | + | + | + |
| **CHZ** | 0.029 | 0.045 | 0.024 | 0.025 | 0.044 | 0.084 | 0.040 | 0.026 | 0.040 | 0.016 | 0.059 | 0.023 | 0.014 | 0.000 | + | + | + |
| **ZW** | 0.020 | 0.031 | 0.017 | 0.018 | 0.012 | 0.054 | 0.022 | 0.013 | **0.011** | 0.043 | 0.039 | 0.020 | 0.076 | 0.031 | 0.000 | + | + |
| **MAS** | 0.029 | 0.047 | 0.035 | 0.037 | 0.022 | 0.02 | 0.045 | 0.027 | 0.013 | 0.046 | 0.032 | 0.034 | 0.091 | 0.049 | 0.028 | 0.000 | + |
| **SQ** | 0.024 | 0.035 | 0.035 | 0.034 | 0.014 | 0.024 | 0.052 | 0.033 | 0.026 | 0.047 | 0.062 | 0.036 | 0.084 | 0.046 | 0.018 | 0.019 | 0.000 |
