# Supplementary material for: Fine-scale genetic structure and wolbachia infection of aedes albopictus (Diptera: Culicidae) in Nanjing city, China
Source: Front Genet. 2022 Aug 30;13:827655. doi: 10.3389/fgene.2022.827655 (PMC9468874; doi:10.3389/fgene.2022.827655)
Supplement: Supplementary file 4 [file Table3.DOCX]

**Additional file 4: Table S3. Heterozygosity tests of all 17 *Ae. albopictus* populations based on the stepwise mutation model (S.M.M.) and two-phase model (T.P.M.)**

| Nanjing City | | ZSL | MYJ | YHXC | QGXC | RHJY | NQSQ | NQBY | HAJY | JMJY | TPSQ | HLXY | HSZY | LV | CHZ | ZW | MAS | SQ |
| --- | --- | --- | --- | --- | --- | --- | --- | --- | --- | --- | --- | --- | --- | --- | --- | --- | --- | --- |
| S.M.M. | He < Heq | 7 | 4 | 4 | 5 | 5 | 4 | 5 | 7 | 3 | 5 | 5 | 6 | 3 | 6 | 6 | 4 | 5 |
|  | He > Heq | 2 | 4 | 4 | 3 | 3 | 5 | 2 | 2 | 5 | 4 | 3 | 2 | 5 | 3 | 2 | 5 | 3 |
|  | *P* (He < Heq) | 0.027 | 0.430 | 0.441 | 0.170 | 0.214 | 0.532 | 0.130 | 0.026 | 0.574 | 0.321 | 0.246 | 0.049 | 0.544 | 0.103 | 0.081 | 0.582 | 0.214 |
| T.P.M. | He < Heq | 7 | 6 | 7 | 6 | 5 | 8 | 5 | 7 | 5 | 8 | 5 | 8 | 6 | 7 | 6 | 6 | 6 |
|  | He > Heq | 2 | 2 | 1 | 2 | 3 | 1 | 2 | 2 | 3 | 1 | 3 | 0 | 2 | 2 | 2 | 3 | 2 |
|  | *P* (He < Heq) | 0.039 | 0.593 | 0.010 | 0.060 | 0.180 | 0.007 | 0.110 | 0.036 | 0.193 | 0.005 | 0.250 | 0.001 | 0.053 | 0.031 | 0.078 | 0.191 | 0.073 |
